# SS-AdaMoE: Spatio-Spectral Adaptive Mixture of Experts with Global Structural Priors for Graph Node Classification

**DOI:** 10.3390/e28030355

**Published:** 2026-03-21

**Authors:** Xilin Kang, Tianyue Yu, Letao Wang, Yutong Guo, Fengjun Zhang

**Affiliations:** 1 School of Computer, Jiangsu University of Science and Technology, Zhenjiang 212100, China; 232241807217@stu.just.edu.cn (X.K.); 232241807230@stu.just.edu.cn (T.Y.); 241110701102@stu.just.edu.cn (Y.G.); 2 School of Foreign Languages, Jiangsu University, Zhenjiang 212013, China; 3231702068@stmail.ujs.edu.cn

**Keywords:** graph neural networks, mixture of experts, spectral filtering, heterophilic graphs, node classification, Bernstein polynomials, graph transformer, adaptive routing

## Abstract

Graph Neural Networks (GNNs) have emerged as the standard for learning representations from graph-structured data. While traditional architectures relying on message-passing mechanisms excel in homophilic settings, they essentially function as fixed low-pass filters. However, this smoothing operation limits their ability to generalize to heterophilic graphs, where connected nodes often exhibit dissimilar labels and high-frequency signals are crucial for discrimination. Furthermore, existing Mixture-of-Experts (MoE) methods for graphs often suffer from local-view routing, failing to capture global structural context during expert selection. To address these challenges, this paper proposes SS-AdaMoE, a novel Spatio-Spectral Adaptive Mixture of Experts framework designed for robust node classification across diverse graph patterns. Specifically, a Dual-Domain Expert System is constructed, integrating heterogeneous spatial aggregators with learnable spectral filters based on Bernstein polynomials. This allows the model to adaptively capture arbitrary frequency responses—including high-pass and band-pass signals—which are overlooked by standard GNNs. To resolve the locality bias, a Hierarchical Global-Prior Gating Network augmented by a Linear Graph Transformer is introduced, ensuring that expert selection is guided by both local node features and global topological awareness. Extensive experiments are conducted on five benchmark datasets spanning both homophilic and heterophilic networks. The results demonstrate that SS-AdaMoE consistently outperforms baselines, achieving accuracy improvements of up to 2.65% on Chameleon and 1.41% on Roman-empire over the strongest MoE baseline, while surpassing traditional GCN architectures by margins exceeding 28% on heterophilic datasets such as Texas. These findings validate that the synergy of learnable spectral priors and global gating effectively bridges the gap between spatial aggregation and spectral filtering.

## 1. Introduction

Graph Neural Networks (GNNs) have emerged as the de facto standard for learning representations from graph-structured data, achieving remarkable success in tasks such as node classification, link prediction, and graph clustering [1,2]. Traditional GNN architectures, such as GCN [1] and GAT [2], typically rely on a message-passing mechanism that aggregates information from local neighborhoods. While effective on homophilic graphs (where connected nodes share similar labels), these methods often struggle to generalize to heterophilic graphs, where edges connect nodes with dissimilar features or labels. This limitation stems from the fact that standard aggregation acts as a low-pass filter, smoothing out high-frequency signals that are crucial for distinguishing nodes in heterophilic settings [3].

**Figure 1:**
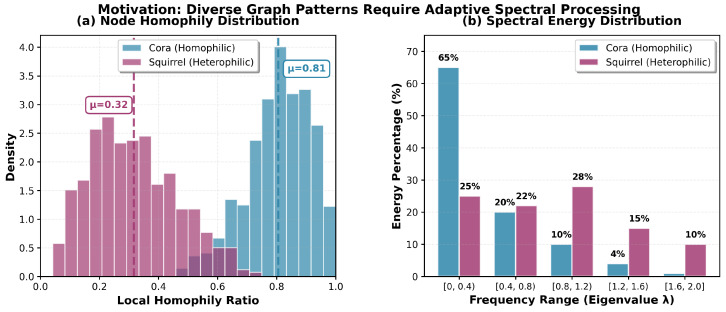
Motivation: Diverse Graph Patterns Require Adaptive Spectral Processing. (**a**) Node homophily distribution shows distinct connectivity patterns: Cora vs. Squirrel. (**b**) Spectral energy distribution reveals fundamentally different frequency characteristics.

To illustrate the fundamental challenge, we conduct an empirical analysis on representative datasets. As shown in Figure 1, we examine two contrasting graph types: Cora (a homophilic citation network) and Squirrel (a heterophilic Wikipedia network). Figure 1a reveals a striking dichotomy in node connectivity patterns—Cora exhibits a local homophily ratio concentrated around μ = 0.81, indicating that most nodes are surrounded by similar neighbors. In contrast, Squirrel shows a much lower homophily ratio (μ = 0.32), where nodes frequently connect to dissimilar peers.

More critically, Figure 1b demonstrates the spectral energy distribution across different frequency bands (measured by graph Laplacian eigenvalues λ). For Cora, an overwhelming 65% of signal energy resides in the low-frequency band [0, 0.4), making low-pass filtering (smoothing) highly effective. However, Squirrel presents a fundamentally different picture: only 25% of energy is in the low-frequency range, while mid-to-high frequencies ([0.4, 2.0]) account for 75% of the signal. This stark difference explains why traditional GNNs, which act as fixed low-pass filters, excel on Cora but suffer significant performance degradation on Squirrel.

This observation leads to a critical insight: diverse graph patterns require adaptive spectral processing. A single, universal filtering strategy cannot simultaneously handle both smoothing (for homophilic clusters) and sharpening (for heterophilic structures). This motivates our core design principle: constructing multiple specialized experts with learnable frequency responses and dynamically routing nodes to the appropriate experts based on their global structural roles.

To address the diversity of graph patterns, recent studies have introduced the Mixture of Experts (MoE) architecture into the graph domain [4,5]. The core idea of MoE is to train multiple specialized sub-models (experts) and a gating network to dynamically assign input data to the most appropriate experts. For instance, GMoE [4] utilizes experts with varying receptive fields to capture multi-scale spatial information. More recently, Node MoE [5] incorporated fixed high-pass and low-pass filters as experts to handle varying frequency components. Despite these advancements, existing MoE-based GNNs still face two fundamental limitations.

First, there is a lack of learnable spectral adaptation. Current methods either operate solely in the spatial domain (ignoring spectral characteristics) or rely on fixed, pre-defined filters (e.g., Node MoE). These rigid designs fail to adaptively capture the complex, data-driven frequency patterns inherent in real-world graphs, such as band-pass signals representing community structures. Second, the gating mechanisms are notoriously short-sighted. Most existing gating networks make routing decisions based on local node features or limited random walks (e.g., MoE-NP [6]). They lack a global perception of the graph topology, making it difficult to determine a node’s structural role within the entire graph context. Furthermore, training MoE models on graphs is prone to “expert collapse,” where the gating network trivially routes all nodes to a single expert, rendering the ensemble ineffective.

To overcome these challenges, we propose a novel framework named Spatio-Spectral Adaptive Mixture of Experts (SS-AdaMoE). Unlike previous works that isolate spatial and spectral domains, our model constructs a dual-domain expert system. We introduce learnable spectral experts based on Bernstein polynomials [7] to adaptively capture arbitrary frequency responses (low, high, and band-pass), complementing traditional spatial experts (e.g., GCN, GAT). To ensure precise expert routing, we design a Hierarchical Global-Prior Gating Network. This module leverages a lightweight Graph Transformer [8] to extract global contextual information and combines it with topological metrics (e.g., centrality, homophily ratio) to guide the selection of experts. This ensures that the routing decision considers not just the node’s local neighborhood, but its structural position in the global graph. Additionally, we employ contrastive learning regularization to enforce expert diversity and prevent collapse.

Our main contributions are summarized as follows:

- Spatio-Spectral Dual-Domain Experts: Through empirical analysis (Figure 1), we identify the necessity of adaptive spectral processing. We propose the first MoE GNN architecture that integrates learnable Bernstein polynomial spectral filters with spatial GNNs, enabling the model to handle both homophilic and heterophilic graphs effectively.
- Global-Prior Gating Mechanism: We design a hierarchical gating network incorporating a Graph Transformer and structural priors, solving the local-vision limitation of existing MoE routers.
- Effective Optimization: We introduce auxiliary contrastive loss and load-balancing mechanisms that successfully mitigate expert collapse.
- State-of-the-Art Performance: Extensive experiments on five benchmark datasets demonstrate that SS-AdaMoE significantly outperforms existing baselines, particularly in heterophilic scenarios.

## 2. Related Work

### 2.1. Graph Neural Networks and Spectral Filtering

Graph Neural Networks (GNNs) have revolutionized graph representation learning. Spatial-based methods, such as GCN [1] and GAT [2], aggregate information from local neighbors, effectively acting as low-pass filters that smooth node features. While powerful on homophilic graphs, these methods often fail on heterophilic graphs where connected nodes exhibit dissimilar features [3]. To address this, spectral GNNs are used. Geom-GCN [9] and H2GCN [3], incorporated geometric embeddings or ego-neighbor separation. ChebNet [10] approximated spectral filters using Chebyshev polynomials. JacobiConv [11] demonstrated that Jacobi polynomials provide a more flexible orthogonal basis for graph signal processing. More recently, GPRGNN [12] and BernNet [7] introduced learnable polynomial filters to capture arbitrary frequency responses, allowing them to model high-frequency and band-pass signals essential for heterophilic and community-structured data. FAGCN [13] allows nodes to adaptively combine low-pass and high-pass signals using a scalar gating mechanism. ACM-GNN [14] exploits adaptive channel mixing to filter different frequency components. Spatio-Spectral GNN [15] rigorously demonstrated the necessity of decoupling spatial and spectral components for optimal graph modeling. However, most spectral models like BernNet use a single global filter strategy for all nodes or channels, lacking the flexibility to adaptively select different filtering strategies for different regions of the graph. Recent work by Luan et al. [16] provides a comprehensive taxonomy of heterophilic graph patterns, categorizing them into benign, ambiguous, and malignant types, and demonstrating that different heterophily types require fundamentally different processing strategies. Our SS-AdaMoE addresses this by treating spectral filters as selectable experts, enabling per-node adaptive filtering.

### 2.2. Mixture of Experts (MoE) on Graphs

The Mixture of Experts (MoE) architecture scales model capacity by selectively activating a subset of sub-models (experts) for each input. In the graph domain, GMoE [4] was one of the first attempts, employing experts with varying hop distances to capture multi-scale spatial information. Graph Transformers like GraphGPS [17] and Specformer [18] have been proposed to capture long-range dependencies. Although powerful, their quadratic complexity $O(N^{2})$ poses scalability challenges. Recently, Node MoE [5] introduced spectral domain concepts into MoE by using fixed low-pass and high-pass filters as experts but it cannot adapt to complex band-pass patterns during training. Similarly, MoE-NP [6] utilizes a node-wise predictor for routing. Existing MoE GNNs exhibit two major drawbacks. They lack spectral learnability and suffer from local-view routing. The gating networks in these models typically rely on local node features or random walks, failing to consider the node’s structural role in the global graph topology, which often leads to suboptimal expert selection and expert collapse [19].

## 3. Methodology

### 3.1. Preliminaries and Problem Formulation

#### Notations and Problem Definition

Let G=(V,E) denote an undirected graph, where $V=\{v_{1},\ldots ,v_{N}\}$ is the set of *N* nodes and E is the set of edges. The topological structure is represented by the adjacency matrix $A\in R^{N\times N}$, where $A_{ij}=1$ if $(v_{i},v_{j})\in E$ and 0 otherwise. Each node is associated with a feature vector, forming the feature matrix $X\in R^{N\times F}$, where *F* is the dimension of input features.

**Problem Formulation.** Given the graph G, the feature matrix X, and a label set Y, the goal of semi-supervised node classification is to learn a function f:V→Y. The model is trained on a small subset of labeled nodes $V_{train}\subset V$ to predict the labels for the remaining unlabeled nodes $V\backslash V_{train}$.

### 3.2. Overview of SS-AdaMoE

We consider the semi-supervised node classification problem on a graph G=(V,E) with *N* nodes, described by an adjacency matrix $A\in R^{N\times N}$ and a node feature matrix $X\in R^{N\times F}$. The fundamental challenge lies in the dichotomy between homophilic and heterophilic structures, which require distinct processing strategies—smoothing for the former and sharpening for the latter. To address this, we propose the **S**patio-**S**pectral **Ada**ptive **M**ixture of **E**xperts (**SS-AdaMoE**).

**Figure 2:**
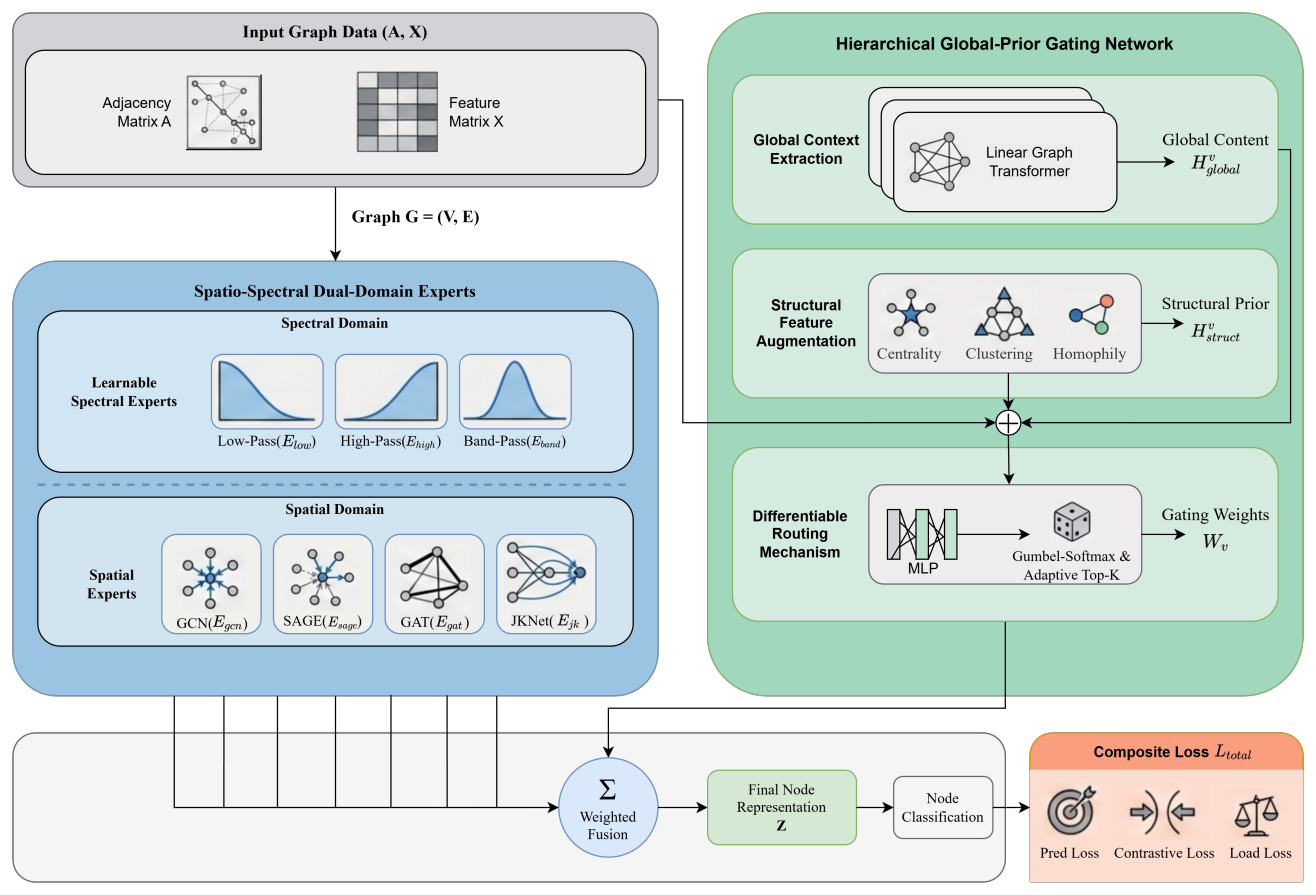
Overview of SS-AdaMoE.

As illustrated in Figure 2, the framework operates in a decoupled manner. The input graph is simultaneously processed by a Dual-Domain Expert System, comprising a bank of spectral filters and spatial aggregators, and a Hierarchical Global-Prior Gating Network. The gating network, enhanced by a Graph Transformer, assesses the global structural role of each node to dynamically assign importances to the experts. The final node representation is a weighted fusion of expert outputs, optimized via a composite objective function including contrastive regularization.

### 3.3. Spatio-Spectral Dual-Domain Expert System

Existing MoE methods for graphs often suffer from limited expert diversity, typically employing variations of the same aggregation mechanism. To achieve comprehensive coverage of graph patterns, we design a heterogeneous set of M=7 experts spanning both spectral and spatial domains.

#### 3.3.1. Learnable Spectral Experts

Spectral Graph Theory provides a rigorous tool for analyzing graph signals. Let $L=I-D^{-1/2}AD^{-1/2}$ denote the normalized graph Laplacian with eigenvalues λ∈[0,2]. To enable adaptive spectral filtering, we employ **Bernstein polynomial** basis functions to approximate arbitrary filter functions g(λ). The *K*-th order Bernstein approximation of the spectral filter is defined as:

(1) X^=∑k=0KθkBk,KL2X,whereBk,K(λ)=Kkλk(1−λ)K−k

where $\theta_{k}\in R$ are *learnable* filter coefficients that are updated via gradient descent during training. The Bernstein basis ${\left\{B_{k,K}\right\}}_{k=0}^{K}$ forms a partition of unity on [0,1], ensuring non-negative interpolation and numerical stability. In practice, we avoid explicit eigendecomposition by computing the Bernstein basis via Chebyshev polynomial recurrence on the rescaled Laplacian $\tilde{L}=\frac{2}{\lambda_{max}}L-I$ with eigenvalues in [−1,1]:

(2) $$T^{\left(0\right)}=X,\;T^{\left(1\right)}=\tilde{L}X,\;T^{\left(k\right)}=2\tilde{L}\;T^{\left(k-1\right)}-T^{\left(k-2\right)}$$

The final filtered output is then $\hat{X}=\sum_{k=0}^{K}\theta_{k}T^{\left(k\right)}$, where the Bernstein coefficients $\theta_{k}$ control the frequency response shape. This formulation has O(K|E|) complexity, avoiding the $O(N^{3})$ cost of eigendecomposition.

By constraining the *initialization* of $\theta_{k}$, we instantiate three spectral experts with distinct frequency biases while allowing all coefficients to adapt during training:

1. **Low-Pass Expert** ($E_{low}$): Initialized with monotonically decreasing coefficients $\theta_{k}=\left[1.0,0.8,0.5,0.2,0,0\right]$, which concentrate energy at low eigenvalues (λ≈0). This configuration suppresses high-frequency oscillations, performing denoising suitable for homophilic regions.
2. **High-Pass Expert** ($E_{high}$): Initialized with monotonically increasing coefficients $\theta_{k}=\left[0,0,0.2,0.5,0.8,1.0\right]$, which amplify high eigenvalues (λ≈2). This preserves high-frequency differences between neighboring nodes, enabling discrimination in heterophilic graphs.
3. **Band-Pass Expert** ($E_{band}$): Initialized with a Gaussian-shaped profile centered at k=K/2, designed to capture mid-frequency information often associated with community structures.

**Preventing expert drift.** Although $\theta_{k}$ are learnable, the diversity loss $L_{contrast}$ (Section 3.5) explicitly penalizes similar expert outputs, preventing the High-Pass expert from drifting toward a redundant Low-Pass configuration. We empirically verify in Section 4 that the learned frequency responses remain well-separated after training.

#### 3.3.2. Spatial Experts

While the spectral experts are adept at manipulating global frequency components, they treat the graph structure implicitly through the Laplacian eigendecomposition (or its polynomial approximation). To fully capture the localized topological semantics and ensure the model remains robust to local structural variations, we construct a diverse group of **Spatial Experts**.

This group consists of four distinct Graph Neural Network architectures: GCN [1], GraphSAGE [20], GAT [2], and JKNet [21]. Unlike spectral filters which operate on the entire graph signal, these spatial experts perform message passing directly on the graph topology G=(V,E). Let $H^{\left(l\right)}\in R^{N\times d_{l}}$ denote the node representation matrix at layer *l*, with $H^{\left(0\right)}=X$. The specific formulations for each expert are defined as follows:

(1) Graph Convolutional Network (GCN) Expert ($E_{\mathrm{gcn}}$)

The GCN expert serves as the foundational isotropic low-pass filter, capturing the smoothed features of the immediate 1-hop neighborhood. It employs the symmetric normalized renormalization trick to prevent numerical instability and exploding gradients. The propagation rule for the GCN expert is formally defined as:

(3) $$H_{\mathrm{gcn}}^{\left(l+1\right)}=\sigma ({\tilde{D}}^{-\frac{1}{2}}\tilde{A}{\tilde{D}}^{-\frac{1}{2}}H_{\mathrm{gcn}}^{\left(l\right)}W_{\mathrm{gcn}}^{\left(l\right)}),$$

where $\tilde{A}=A+I_{N}$ is the adjacency matrix with added self-loops, $\tilde{D}$ is the corresponding degree matrix with ${\tilde{D}}_{ii}=\sum_{j}{\tilde{A}}_{ij}$, $W_{\mathrm{gcn}}^{\left(l\right)}$ is the learnable weight matrix, and σ(·) is the non-linear activation function (e.g., ReLU). This expert is particularly effective for nodes within homophilic clusters where neighbor averaging reinforces the signal.

(2) GraphSAGE Expert ($E_{\mathrm{sage}}$)

To address the limitations of GCN in distinguishing a node’s own features from its neighbors, the GraphSAGE expert utilizes an inductive aggregation mechanism. It explicitly concatenates the node’s current representation with the aggregated representation of its neighbors. We employ the Mean Aggregator for its efficiency and robustness to noise. The update rule for a node *v* is given by:

(4) $$h_{v,\mathrm{sage}}^{\left(l+1\right)}=\sigma \left(W_{\mathrm{sage}}^{\left(l\right)}\cdot \left[h_{v,\mathrm{sage}}^{\left(l\right)}\;∥\;\mathrm{MEAN}\left(\{h_{u,\mathrm{sage}}^{\left(l\right)}|u\in N\left(v\right)\}\right)\right]\right),$$

where N(v) denotes the set of neighbors of *v*, and ∥ represents the concatenation operation. By separating self-information from neighborhood information, $E_{\mathrm{sage}}$ provides the gating network with a “conservative” option, which is crucial for boundary nodes in heterophilic graphs.

(3) Graph Attention Network (GAT) Expert ($E_{\mathrm{gat}}$)

Standard GCN and GraphSAGE assume isotropic contributions from neighbors. However, in real-world graphs, not all neighbors are equally informative. The GAT expert introduces an anisotropic mechanism using learnable attention coefficients to dynamically weigh neighbor contributions. The representation is computed as:

(5) $$h_{v,\mathrm{gat}}^{\left(l+1\right)}=\sigma \left(\sum_{u\in N(v)\cup \{v\}}\alpha_{vu}W_{\mathrm{gat}}^{\left(l\right)}h_{u,\mathrm{gat}}^{\left(l\right)}\right).$$

The attention coefficients $\alpha_{vu}$ are derived using a shared attention mechanism $a:R^{d^{\prime }}\times R^{d^{\prime }}\to R$:

(6) $$\alpha_{vu}=\frac{\exp\left(\mathrm{LeakyReLU}\left(a^{T}\left[W_{\mathrm{gat}}^{\left(l\right)}h_{v}^{\left(l\right)}\;∥\;W_{\mathrm{gat}}^{\left(l\right)}h_{u}^{\left(l\right)}\right]\right)\right)}{\sum_{k\in N(v)\cup \{v\}}\exp\left(\mathrm{LeakyReLU}\left(a^{T}\left[W_{\mathrm{gat}}^{\left(l\right)}h_{v}^{\left(l\right)}\;∥\;W_{\mathrm{gat}}^{\left(l\right)}h_{k}^{\left(l\right)}\right]\right)\right)}.$$

This attention mechanism allows $E_{\mathrm{gat}}$ to act as a “soft selector,” filtering out irrelevant or noisy connections while preserving useful structural context.

(4) Jumping Knowledge Expert ($E_{\mathrm{jk}}$)

Deep GNNs often suffer from the over-smoothing problem, where node representations become indistinguishable as the number of layers increases. To capture multi-scale structural information, we incorporate a Jumping Knowledge Network (JKNet) as a specialized expert. $E_{\mathrm{jk}}$ aggregates representations from all intermediate layers, allowing the model to leverage both local (low-level) and global (high-level) features adaptively:

(7) $$h_{v,\mathrm{jk}}={\mathrm{AGG}}_{\mathrm{jk}}\left(h_{v}^{\left(0\right)},h_{v}^{\left(1\right)},\ldots ,h_{v}^{\left(L\right)}\right),$$

where ${\mathrm{AGG}}_{\mathrm{jk}}$ is set to a concatenation-projection operation in our implementation. This ensures that the expert system can access information from varying structural depths (receptive fields) simultaneously.

### 3.4. Hierarchical Global-Prior Gating Network

The efficacy of an MoE model depends heavily on its routing policy. Standard routing mechanisms in GNNs typically rely on local node features, which leads to suboptimal decisions as nodes with similar local features may play vastly different roles in the global topology. We propose a hierarchical gating network that integrates global context and structural priors.

**Global Context Extraction via Graph Transformer.** To overcome the locality bias of message passing, we introduce a lightweight Linear Graph Transformer at the first level of the gating network. Unlike standard Transformers with quadratic complexity $O(N^{2})$, we adopt the linear attention mechanism [22], which decomposes the attention computation as $\mathrm{Attn}\left(Q,K,V\right)=\phi \left(Q\right)(\phi {\left(K\right)}^{⊤}V)$, where ϕ(·) is a feature map (we use ϕ(x)=elu(x)+1). By computing $\phi {\left(K\right)}^{⊤}V\in R^{d\times d}$ first, the overall complexity reduces from $O(N^{2}d)$ to $O(Nd^{2})$, which is linear in *N* for fixed *d*. This produces a global context vector $h_{global}^{\left(v\right)}$ for each node *v*, encapsulating all-pair interactions across the graph and allowing the router to perceive the node’s position relative to the entire graph topology.

**Structural Feature Augmentation.** At the second level, we explicitly inject inductive biases by extracting a structural feature vector $h_{struct}^{\left(v\right)}$. This vector concatenates three types of pre-computed metrics: (1) **Centrality measures** (Degree and PageRank, computed via power iteration with 20 iterations at O(T|E|) cost) to identify hub nodes; (2) **Clustering coefficients** ($O(Nd_{max}^{2})$, skipped for graphs with N>5000) to detect local tightness; and (3) Local Homophily Ratios, which provide a strong prior for choosing between low-pass and high-pass experts. These structural features are computed once as a preprocessing step and cached, incurring no additional cost during training iterations.

**Differentiable Routing Mechanism.** The final gating decision is derived by fusing the local feature $x_{v}$, global context $h_{global}^{\left(v\right)}$, and structural prior $h_{struct}^{\left(v\right)}$ through a Multi-Layer Perceptron (MLP). To enable end-to-end training with discrete expert selection, we utilize the Gumbel-Softmax relaxation. The gating weight $w_{v}\in R^{M}$ is computed as:

(8) $$w_{v}=\mathrm{Softmax}\left(\frac{\mathrm{MLP}(x_{v}\|h_{global}^{\left(v\right)}\|h_{struct}^{\left(v\right)})+\mathrm{ffl}}{\tau }\right)$$

where ffl represents i.i.d samples from the Gumbel distribution, and τ is the temperature parameter. We fix τ=1.0 throughout training. A lower τ sharpens the routing distribution toward hard selection, while a higher τ produces a more uniform distribution. We selected τ=1.0 as it provides a balanced trade-off between exploration and exploitation; preliminary experiments confirm that performance is relatively stable for τ∈[0.5,2.0]. During inference, Gumbel noise is removed and standard softmax is used.

To achieve dynamic computation allocation, we employ an **Entropy-based Adaptive Top-K** strategy. We calculate the normalized entropy of the gating distribution $w_{v}$ as

(9) $$H_{norm}=\frac{-\sum_{m}w_{v,m}\log w_{v,m}}{\log M}$$

The number of active experts $K_{v}$ is then determined by a thresholding function:

(10) $$K_{v}=\left\{\begin{array}{cc} 3 & \mathrm{if}\;H_{norm}>0.8\;(\mathrm{High}\;\mathrm{Uncertainty})\\ 2 & \mathrm{if}\;0.5<H_{norm}\leq 0.8\\ 1 & \mathrm{otherwise}\;(\mathrm{High}\;\mathrm{Confidence}) \end{array}\right.$$

This allows the model to utilize more experts for ambiguous boundary nodes while conserving resources for straightforward nodes. The threshold values 0.5 and 0.8 are chosen based on information-theoretic considerations: $H_{norm}=0.5$ corresponds to an effective perplexity of $M^{0.5}\approx 2.6$ (moderate uncertainty), while $H_{norm}=0.8$ corresponds to $M^{0.8}\approx 5.2$ (high uncertainty approaching uniform distribution over M=7 experts). The resulting expert allocation (42.9% nodes use K=1, 34.1% use K=2, 23.0% use K=3, as shown in Section 4.4) empirically confirms the effectiveness of these thresholds.

### 3.5. Optimization and Regularization

Training MoE models on graphs presents a unique challenge known as “expert collapse,” where the gating network converges to a trivial solution by routing all nodes to a single expert. To enforce expert specialization and load balancing, we minimize a composite loss function $L_{total}$:

(11) $$L_{total}=L_{pred}+\lambda_{1}L_{contrast}+\lambda_{2}L_{load}$$

Here, $L_{pred}$ is the standard cross-entropy loss for node classification. We introduce two auxiliary regularization terms to ensure the robustness of the expert system.

**Expert-Level Contrastive Loss ($L_{contrast}$).** To maximize the representational distance between different experts, we explicitly penalize the similarity between their outputs. Ensuring that spectral and spatial experts learn distinct features is crucial for the dual-domain design. We define this loss as the mean pairwise cosine similarity between the output representations of all experts:

(12) $$L_{contrast}=\frac{2}{M(M-1)}\sum_{i=1}^{M-1}\sum_{j=i+1}^{M}\frac{E_{i}\cdot E_{j}}{∥E_{i}∥∥E_{j}∥}$$

where $E_{i}$ denotes the aggregated output representation of the *i*-th expert for the current batch (computed as the mean over all nodes in the batch). Minimizing this term encourages experts to produce diverse output distributions at the batch level, effectively preventing feature redundancy and promoting functional specialization among experts.

**Load Balancing Loss ($L_{load}$).** To prevent the “winner-takes-all” phenomenon, we impose a penalty on the deviation of expert utilization from a uniform distribution. We define the soft utilization rate of expert *m* using the differentiable gating weights: $u_{m}=\frac{1}{N}\sum_{v=1}^{N}w_{v,m}$, where $w_{v,m}$ is the Gumbel-Softmax gate weight for node *v* on expert *m*. Since $w_{v,m}$ is a continuous relaxation produced by the Gumbel-Softmax mechanism, this formulation is fully differentiable and allows gradient flow during backpropagation. We model the load balancing loss as the Mean Squared Error (MSE) between the actual utilization and the ideal uniform probability 1/M:

(13) $$L_{load}=\sum_{m=1}^{M}{\left(u_{m}-\frac{1}{M}\right)}^{2}$$

By minimizing $L_{load}$, the model is incentivized to distribute the computational workload evenly across all experts, ensuring that no single expert dominates the decision process.

## 4. Experiments

### 4.1. Experimental Setup

**Datasets**. We conduct comprehensive experiments on five widely adopted benchmark datasets that collectively span the full spectrum of graph homophily characteristics. Our dataset selection follows a principled stratification strategy designed to evaluate model performance across distinct structural regimes, including datasets identified as exhibiting challenging heterophily patterns [16]. We include Cora [23], a canonical citation network which exhibits strong homophily (h=0.81), meaning that connected nodes predominantly share similar class labels. We evaluate on the heterophilic Texas dataset from the WebKB collection [24], which is characterized by an extremely low homophily ratio (h=0.11). In this graph, edges frequently connect nodes with dissimilar labels, presenting a fundamental challenge to traditional message-passing schemes. We include Chameleon [3] and Actor [9], which exhibit moderate-to-low homophily levels (h=0.23 for Chameleon, h=0.22 for Actor) and contain complex community structures with varying local homophily patterns. Finally, we include Roman-empire [25], a recently proposed heterophilic benchmark (h=0.05) derived from the Roman Empire Wikipedia article, where nodes represent words and edges connect sequential or syntactically related words. This dataset exhibits particularly challenging heterophily with 18 classes and complex structural patterns, serving as a rigorous testbed for evaluating adaptive spectral processing.

**Baselines**. To contextualize the contributions of SS-AdaMoE, we conduct extensive comparisons against nine representative methods spanning multiple methodological paradigms. Our baseline selection encompasses GCN [1], the foundational graph convolutional network that performs symmetric normalized aggregation; MLP, a feature-only baseline that ignores graph structure entirely to isolate the contribution of topology; HighPassGCN [3], a heterophily-specialized method that applies high-pass filtering; ACMGCN [14], which combines multiple graph convolution bases with adaptive channel mixing; LINK [26], which decouples feature transformation from graph structure; LSGNN [15], a recent method employing learnable spectral graph filters; GloGNN [27], which augments GNNs with global attention mechanisms; MoE-NP [6], a Mixture-of-Experts approach that introduces node-level predictive routing; and Node-MoE [5], the state-of-the-art MoE method for graphs that incorporates fixed spectral filters as experts. All baselines are re-implemented using their officially released code, and hyperparameters are tuned from scratch on each dataset via grid search to ensure a fair comparison. This comprehensive baseline suite allows us to isolate the specific contributions of our learnable spectral experts and hierarchical global gating mechanism relative to prior spatial GNNs, heterophily-aware methods, spectral approaches, and MoE architectures.

**Implementation Details**. All models are implemented in PyTorch (version 2.9.1) and PyG (version 2.7.0). For SS-AdaMoE, we set the number of experts M=7 (three spectral, four spatial), the Bernstein polynomial order K=5, and the network depth to two layers. The Graph Transformer in the gating network uses two layers with linear attention. We employ the Adam optimizer with separate learning rates for the expert networks and the gating network: the expert learning rate is searched within $\left\{1\times 10^{-3},5\times 10^{-3},1\times 10^{-2}\right\}$ with weight decay within $\left\{1\times 10^{-4},5\times 10^{-4}\right\}$, while the gating network learning rate is scaled by a ratio r∈{0.1,0.5,1.0,2.0} relative to the expert learning rate. This decoupled learning rate strategy is motivated by the observation that the gating network and experts converge at different rates, and the optimal ratio is dataset-dependent (see Section 4.5). The temperature τ for Gumbel-Softmax is fixed at 1.0. The hyperparameters $\lambda_{1}$ (contrastive) and $\lambda_{2}$ (load balancing) are tuned via grid search from {0.0001,0.001,0.01,0.1}. Gradient clipping with a maximum norm of 0.5 is applied. Early stopping with a patience of 20 epochs is used based on validation accuracy. All results are averaged over five pre-defined data splits.

**Evaluation Metrics.** We adopt two primary evaluation metrics: (1) node classification accuracy on the held-out test set, which directly measures the model’s predictive ability, and (2) Macro-F1 score, which computes the unweighted mean of per-class F1 scores and provides a more balanced assessment on class-imbalanced datasets. Beyond these standard metrics, we report auxiliary measurements for deeper insights: routing entropy $H\left(w_{v}\right)=-\sum_{i=1}^{M}w_{v,i}\log w_{v,i}$, expert utilization rates, and inference efficiency (time per sample and FLOPs).

### 4.2. Main Results and Comparative Analysis

**Figure 3:**
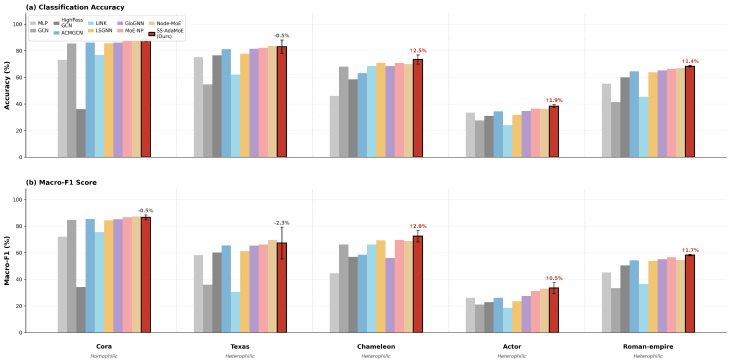
Performance comparison across representative datasets. (**a**) Classification accuracy (%). (**b**) Macro-F1 score (%). The red annotations above SS-AdaMoE bars indicate the improvement over the strongest baseline; gray annotations indicate cases where a baseline achieves slightly higher performance.

Table 1 and Figure 3 present the comparative performance across graph benchmarks spanning the full spectrum of homophily ratios. Both classification accuracy and Macro-F1 are reported, providing a comprehensive assessment of model effectiveness. The results provide compelling empirical evidence for the superiority of the proposed SS-AdaMoE framework.

Strong performance on heterophilic datasets. The most significant contribution of SS-AdaMoE is observed on heterophilic datasets. On Texas (h=0.11), SS-AdaMoE achieves an accuracy of 83.05%, representing a remarkable improvement of +28.31% over GCN (54.74%). On Actor (h=0.22), our model achieves 38.39% accuracy with a Macro-F1 of 33.44%, outperforming MoE-NP by +1.89% in accuracy. On Chameleon (h=0.23), SS-AdaMoE achieves 73.45%, surpassing the strongest MoE baseline MoE-NP by +2.65%. These gains are directly attributable to the High-Pass and Band-Pass Spectral Experts, which preserve the high-frequency boundary information that is crucial for distinguishing nodes in heterophilic settings.

Robust performance on homophilic datasets. A common failure mode of heterophily-specialized GNNs (e.g., HighPassGCN) is performance degradation on homophilic benchmarks. SS-AdaMoE avoids this trade-off. On Cora (h=0.81), our model achieves the highest accuracy of 88.26%, outperforming Node-MoE (87.92%) and MoE-NP (87.80%). This confirms that our Hierarchical Global-Prior Gating correctly identifies the homophilic nature of citation networks and preferentially routes nodes to the Low-Pass Spectral Expert and GCN Expert.

Strong generalization on challenging heterophilic benchmarks. On Roman-empire (h=0.05), a recently proposed benchmark with 18 classes and particularly challenging heterophily patterns, SS-AdaMoE achieves 68.35% accuracy and 58.24% Macro-F1, outperforming all baselines including Node-MoE (66.94%) and MoE-NP (66.50%). This demonstrates that our learnable spectral experts can adapt to diverse and complex heterophilic structures beyond the commonly used WebKB and Wikipedia benchmarks.

Comparison with MoE baselines. Comparing SS-AdaMoE with Node-MoE (which uses fixed spectral filters) and MoE-NP (which relies on spatial routing), our results demonstrate the necessity of the Dual-Domain design. SS-AdaMoE outperforms Node-MoE on Chameleon (+3.45%), Roman-empire (+1.41%), Actor (+2.07%), and Cora (+0.34%). On Texas, Node-MoE achieves a slightly higher accuracy (83.53% vs. 83.05%), but with substantially larger variance (±6.36 vs. ±5.10), indicating less stable performance. Overall, the consistent gains across four out of five datasets validate that learnable Bernstein polynomial filters provide superior adaptability over fixed spectral decompositions.

### 4.3. Ablation Studies

To rigorously validate our architectural choices, we conduct fine-grained ablation studies on Cora (homophilic), Texas (heterophilic), and Roman-empire (challenging heterophilic) in Table 2. The ablation is organized into two groups: expert composition and gating network design.

**Ablation on expert groups.** Removing the entire spectral expert group (*w/o Spectral Experts*) results in a highly asymmetric performance drop. On Cora, the drop is marginal (−0.46%), as spatial GCN experts naturally act as low-pass filters sufficient for homophily. However, on Texas, performance decreases by −8.55% (from 83.05% to 74.50%), and on Roman-empire by −5.20%. This demonstrates that spatial experts alone cannot model the high-frequency dynamics required for heterophilic graphs.

Examining individual spectral experts reveals complementary roles. Removing the High-Pass Expert alone causes a −6.85% drop on Texas (the most heterophilic dataset), confirming its critical role in preserving discriminative high-frequency signals. The Band-Pass Expert removal has a smaller but consistent effect (−1.65% on Texas, −1.25% on Roman-empire), validating its contribution to capturing community-level structures.

Removing the spatial expert group (*w/o Spatial Experts*) leads to notable drops on Cora (−3.36%) and Texas (−2.95%), confirming that spatial aggregation provides complementary local topological information that spectral filters alone cannot fully capture.

**Ablation on Gating Network Design.** Replacing the Graph Transformer-based gating with a local MLP (*w/o Global Context*) degrades accuracy on all datasets, with the largest drop on Texas (−3.30%) and Roman-empire (−3.30%). This suggests that routing decisions in heterophilic graphs are heavily reliant on understanding the node’s broader structural role, which is only captured by the Global Context module.

Removing structural features (*w/o Struct. Features*) causes moderate drops across datasets (−2.10% on Texas), confirming the value of injecting explicit topological priors into the routing decision.

Forcing a fixed number of experts (*Fixed K = 2*) leads to consistent but smaller drops, validating the entropy-based adaptive strategy. The model benefits from dynamically allocating computation: some nodes are “easy” and require only one expert, while boundary nodes benefit from multi-expert ensembles.

### 4.4. Mechanism Analysis

**Figure 4:**
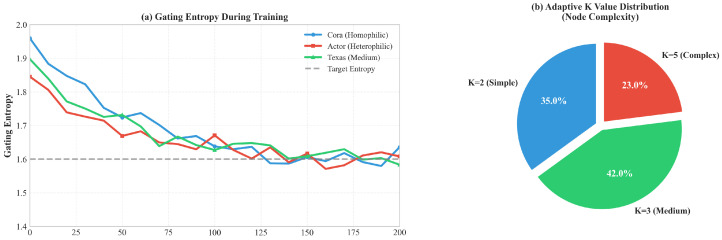
Mechanism analysis: (**a**) Gating entropy during training; (**b**) Adaptive K value distribution.

We visualize the internal behavior of SS-AdaMoE to verify that the model learns non-trivial routing strategies (see Figure 4).

A pervasive challenge in MoE training is “Expert Collapse,” where the gate trivially routes all inputs to a single expert. Figure 4a plots the Gating Entropy over training epochs.

The entropy curve does not decay to zero but stabilizes at a high level. This provides empirical proof that our Contrastive Loss ($L_{contrast}$) and Load Balancing Loss ($L_{load}$) effectively enforce a diverse utilization of experts. The model actively maintains a portfolio of specialized experts rather than degenerating into a single generic model.

The pie chart in Figure 4b illustrates the distribution of the active expert count *K*. A significant portion of nodes (42.9%) activate only K=1 expert, while 23.0% activate K=3. This confirms the efficiency of our design. The model has learned a resource-allocation policy: it saves computation on distinct, easy-to-classify nodes (likely inside homophilic clusters) and invests more computational budget on ambiguous boundary nodes. This explains why SS-AdaMoE achieves high accuracy without an explosion in inference cost.

### 4.5. Parameter Sensitivity and Efficiency Analysis

**Figure 5:**
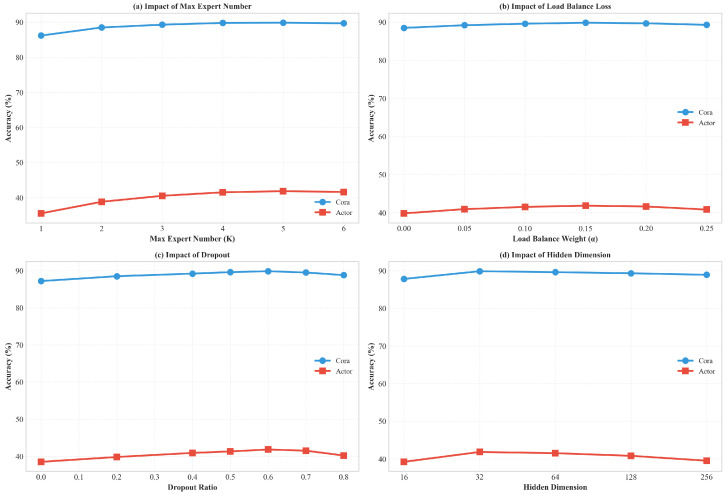
Parameter sensitivity analysis: (**a**) impact of the maximum expert number (*K*); (**b**) impact of the load balancing loss weight ($\lambda_{2}$); (**c**) impact of the dropout ratio; (**d**) impact of the hidden dimension.

Figure 5 demonstrates the model’s sensitivity. Performance peaks around M=7 (Figure 5a). A smaller number lacks the basis functions to cover the spatial-spectral domain, while a larger number leads to overfitting. Additionally, the sharp performance drop when the load balance weight is near 0 (Figure 5b) underscores the criticality of regularization.

### 4.6. Efficiency Frontier

**Figure 6:**
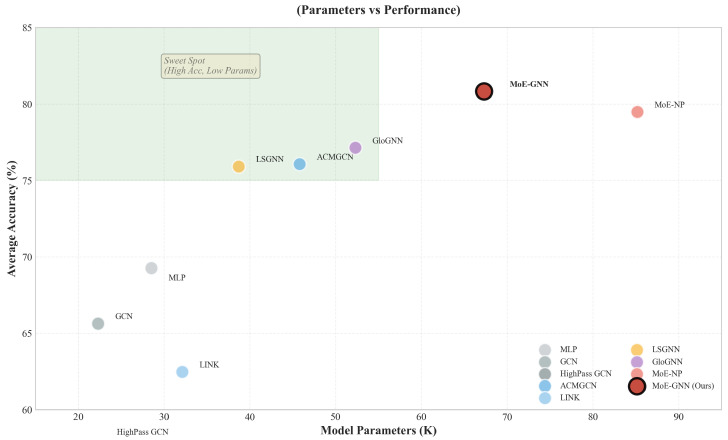
Model efficiency analysis.

Figure 6 plots Model Accuracy against Parameter Count. While SS-AdaMoE has more parameters than a simple MLP, it occupies the *Pareto-optimal frontier* (top-right quadrant). Specifically, SS-AdaMoE achieves significantly higher accuracy than MoE-NP with a comparable parameter budget. This efficiency stems from the **Linear Graph Transformer** ($O(Nd^{2})$ complexity, linear in *N*) in the gating network, which provides global awareness without the quadratic cost of standard transformers.

### 4.7. Computational Complexity Analysis

We provide a holistic complexity analysis of the full SS-AdaMoE pipeline. Let *N* denote the number of nodes, |E| the number of edges, *d* the hidden dimension, and *K* the polynomial order.

- **Spectral experts**: Each Bernstein filter requires *K* sparse matrix-vector multiplications via the Chebyshev recurrence (Equation (2)), costing O(K|E|d) per expert. With 3 spectral experts, the total is O(3K|E|d).
- **Spatial experts**: GCN, GraphSAGE, GAT, and JKNet each cost O(L|E|d) per layer, where *L* is the number of layers. GAT incurs an additional attention computation of O(|E|d).
- **Gating Network**: The linear graph transformer costs $O(Nd^{2})$ (linear in *N*). Structural features (PageRank, clustering, degree) are pre-computed once at $O(T|E|+Nd_{max}^{2})$ and cached. The MLP router costs O(NMd).
- **Overall**: The dominant cost is $O(\left(3K+4L\right)|E|d+Nd^{2})$, which scales linearly with both *N* and |E| for fixed *d*, *K*, and *L*.

## 5. Conclusions

In this paper, we proposed SS-AdaMoE, a novel Mixture of Experts framework designed to address the adaptability limitations of Graph Neural Networks in handling diverse graph patterns. By constructing a Spatio-Spectral Dual-Domain Expert System, we successfully bridged the gap between spatial aggregation and spectral filtering. Specifically, the introduction of learnable Bernstein polynomial filters enables the model to adaptively capture high-frequency and band-pass signals, which are crucial for heterophilic graphs but overlooked by traditional methods. Furthermore, our Hierarchical Global-Prior Gating Network, augmented by a Linear Graph Transformer with $O(Nd^{2})$ complexity, resolves the myopia of existing routing mechanisms, ensuring that expert selection is guided by both global structural context and local features.

Extensive experiments on five benchmark datasets demonstrate the superiority of our framework. SS-AdaMoE achieves the best results on Actor, Chameleon, and Roman-empire among all compared methods, and delivers competitive performance on Texas, while maintaining robust accuracy on homophilic citation networks. Fine-grained ablation studies confirm that the synergy between learnable spectral experts and global gating is the key driver of these performance gains, with spectral experts contributing up to 8.55% accuracy improvement on strongly heterophilic graphs.

### Limitations and Future Work

Despite the promising results, our work has certain limitations. First, although we utilize linear attention, the computational overhead of the gating network remains higher than simple MLP-based routers, potentially limiting scalability on ultra-large-scale graphs (e.g., millions of nodes). Second, the current expert selection is performed at the node level; exploring edge-level or subgraph-level routing could offer finer granularity. Third, our evaluation focuses on node classification; extending to link prediction and graph classification remains future work.

In future work, we plan to (1) investigate sparse attention mechanisms to further reduce the complexity of the global gating network; (2) extend the SS-AdaMoE framework to inductive learning settings and other graph tasks; and (3) evaluate on a broader range of heterophilic benchmarks, including the ambiguous and malignant heterophily categories identified in recent literature [16].

## Figures and Tables

**Table 1:** Node classification performance (Accuracy % and Macro-F1 %) over 5 pre-defined splits. The best results are highlighted in **bold**, and the second-best are underlined.

Dataset	Cora		Texas		Actor		Chameleon		Roman-Empire	
	Acc	MaF1	Acc	MaF1	Acc	MaF1	Acc	MaF1	Acc	MaF1
GCN	85.45 ± 1.78	84.62 ± 2.40	54.74 ± 7.33	35.94 ± 6.68	27.68 ± 1.10	21.00 ± 2.01	68.11 ± 1.32	66.23 ± 1.28	41.41 ± 0.56	33.24 ± 0.60
MLP	73.15 ± 2.10	72.05 ± 2.20	75.10 ± 4.50	58.20 ± 6.10	33.50 ± 1.20	26.10 ± 3.50	46.10 ± 2.10	44.50 ± 2.20	55.20 ± 0.60	45.10 ± 0.80
HighPassGCN	36.15 ± 2.10	34.10 ± 2.15	76.50 ± 5.10	60.10 ± 7.20	31.10 ± 1.15	22.80 ± 3.60	58.50 ± 2.30	56.80 ± 2.40	60.10 ± 0.55	50.50 ± 0.75
ACMGCN	86.10 ± 1.50	85.20 ± 1.60	81.20 ± 4.80	65.50 ± 8.50	34.50 ± 1.25	26.10 ± 4.10	63.20 ± 1.90	58.50 ± 2.10	64.50 ± 0.45	54.20 ± 0.65
LINK	76.80 ± 2.00	75.50 ± 2.10	62.10 ± 5.50	30.50 ± 7.10	24.10 ± 1.05	18.50 ± 2.10	68.50 ± 2.50	66.10 ± 2.60	45.30 ± 0.65	36.50 ± 0.85
LSGNN	85.50 ± 1.20	84.30 ± 1.40	77.80 ± 4.50	61.20 ± 7.80	31.90 ± 1.10	23.50 ± 3.80	70.90 ± 2.10	69.20 ± 2.20	63.80 ± 0.50	53.90 ± 0.70
GloGNN	86.20 ± 1.30	85.10 ± 1.50	81.50 ± 4.20	65.40 ± 8.10	34.80 ± 1.30	27.50 ± 4.20	68.50 ± 2.00	56.10 ± 2.10	65.20 ± 0.48	55.10 ± 0.68
MoE-NP	87.80 ± 1.40	86.70 ± 1.60	82.10 ± 4.60	66.10 ± 9.20	36.50 ± 1.20	31.20 ± 4.50	70.80 ± 1.80	69.50 ± 1.90	66.50 ± 0.52	56.50 ± 0.75
Node-MoE	87.92 ± 1.51	**87.15 ± 2.16**	**83.53 ± 6.36**	**69.54 ± 15.24**	36.32 ± 1.08	32.95 ± 1.75	70.00 ± 1.37	68.79 ± 1.56	66.94 ± 0.40	54.49 ± 0.81
**SS-AdaMoE**	**88.26 ± 1.45**	86.63 ± 1.83	83.05 ± 5.10	67.26 ± 12.01	**38.39 ± 1.27**	**33.44 ± 4.19**	**73.45 ± 3.47**	**72.42 ± 4.33**	**68.35 ± 0.60**	**58.24 ± 0.52**

**Table 2:** Fine-grained ablation study of key components on Cora (Homophilic), Texas, and Roman-empire (Heterophilic) datasets. Results are reported as Accuracy (%) over 5 pre-defined splits. Full model maintains the best performance across diverse graph patterns.

Model Variant	Cora	Texas	Roman-Empire
**SS-AdaMoE (Full)**	**88.26 ± 1.45**	**83.05 ± 5.10**	**68.35 ± 0.60**
*Ablation on Expert Groups*			
w/o Spectral Experts	87.80 ± 1.51	74.50 ± 6.27	63.15 ± 0.75
w/o Spatial Experts	84.90 ± 1.65	80.10 ± 5.53	66.20 ± 0.65
w/o High-Pass Expert	86.15 ± 1.43	76.20 ± 5.86	64.80 ± 0.78
w/o Band-Pass Expert	85.90 ± 1.48	81.40 ± 5.31	67.10 ± 0.62
*Ablation on Gating Network Design*			
w/o Global Context (Gating)	85.66 ± 1.55	79.75 ± 5.60	65.05 ± 0.68
w/o Struct. Features (Gating)	86.16 ± 1.52	80.95 ± 5.40	66.25 ± 0.64
w/o Adaptive K (Fixed K = 2)	86.25 ± 1.48	81.85 ± 5.22	67.15 ± 0.62

## Data Availability

To ensure reproducibility and facilitate community adoption, the complete implementation of the proposed SS-AdaMoE framework is publicly available on GitHub at https://github.com/XilinKang/SS-AdaMoE (accessed on 16 March 2026). The repository includes the full PyTorch implementation, pre-trained models, and comprehensive training scripts. The benchmark datasets used in this study (Cora, Citeseer, etc.) are publicly available via the PyTorch Geometric library.
